# rpL3 promotes the apoptosis of p53 mutated lung cancer cells by down-regulating CBS and NFκB upon 5-FU treatment

**DOI:** 10.1038/srep38369

**Published:** 2016-12-07

**Authors:** Annapina Russo, Assunta Saide, Roberta Cagliani, Monica Cantile, Gerardo Botti, Giulia Russo

**Affiliations:** 1 Department of Pharmacy, University of Naples “Federico II”, Via Domenico Montesano 49, 80131 Naples, Italy; 2 Department of Pathology, Istituto Nazionale Tumori “Fondazione Pascale”-IRCCS via Mariano Semmola, Napoli 80131, Italy

## Abstract

5-FU is a chemotherapy drug commonly used for the treatment of human cancers; however drug resistance represents a major challenge for its clinical application. In the present study, we reporte that rpL3 induced by 5-FU treatment in Calu-6 cells represses CBS transcription and reduces CBS protein stability leading to a decrease of CBS protein levels. rpL3 also regulates negatively the activation of NFκB by preventing NFκB nuclear translocation through IκB-α up-regulation. Furthermore, we demonstrate that rpL3 significantly enhances the apoptosis of 5-FU treated Calu-6 cells promoting the overexpression of the pro-apoptotic proteins Bax and the inhibition of the anti-apoptotic protein Bcl-2. We finally demonstrate that rpL3 potentiates 5-FU efficacy inhibiting cell migration and invasion. Our results suggest that combination of rpL3 and 5-FU is a promising strategy for chemotherapy of lung cancers lacking functional p53 that are resistant to 5-FU.

5-FU has been widely used against a variety of cancers including breast, skin, colorectal, liver, pancreatic and lung cancers^1^. The main mechanism responsible of 5-Fluorouracil (5-FU) activity is the inhibition of thymidylate synthase (TS) and subsequent incorporation of 5-FU metabolites into RNA and DNA^2^. It has been demonstrated that some ribosomal proteins (rp) are critical players in 5-FU treatment of cancer cells. Specifically, 5-FU triggers nucleolar stress and consequently a subset of rp including rpL5, rpL11 and rpL23 are released from ribosome to activate p53 by inhibiting MDM2 pathway^3^. In addition, we recently identified a new p53-independent but still rp-dependent molecular pathway activated in cell response to drug treatment. In particular, we demonstrated that human rpL3 acts as stress sensing molecule essential for cancer cell response to ribosomal stress caused by 5-FU and oxaliplatin (L-OHP) in colon and lung cancer cells lacking active p53^4^. However, the clinical use of 5-FU is limited by drug resistance. In order to develop new strategies to increase 5-FU anticancer activity is important to identify novel genes involved in the molecular signalling pathways activated by 5-FU. These genes could offer new targets for chemotherapy or predictive biomarkers of response to 5-FU-based chemotherapy. Emerging evidences are accumulating regarding a crucial role of cystathionine-*β*-synthase (CBS) in promoting cellular bioenergetics, proliferation and migration in tumors. CBS is one of three principal enzymes involved in the biosynthesis of H_2_S in various mammalian cells and tissues^5^. Recent reports showed high expression levels of CBS in colon^6^, ovarian^7^, prostate^8^ and breast cancer cells^9^. The functional role of changes in the levels of CBS in other types of cancer has not been explored yet^5^ To our knowledge studies in lung have not been reported. CBS catalyzes the synthesis of cystathionine in the trans-sulfuration pathway and its expression is tightly regulated because of its critical role in antioxidant and methylation metabolism. Intracellular amounts of CBS are regulated by transcriptional and epigenetic mechanisms^10^. CBS activity is also regulated by post-translational modifications through a small ubiquitin-like modifier protein which is correlated with the localization of CBS in the nucleus leading to diminished catalytic activity. We have recently demonstrated that CBS can be phosphorylated in a PKG-dependent manner at Ser227 and this event lead to an increased catalytic activity^11^,^12^. Pharmacological inhibition or genetic silencing of CBS is associated with antitumor effects *in vitro* and *in vivo*, and enhances the efficacy of the currently used anticancer drugs as L-OHP^13^.

Several evidence demonstrate that NFκB is constitutively activated in a variety of solid tumors including lung cancer^14^. Activation of NFκB occurs by release from the IκB molecules or by cleavage of the inhibitory ankyrin repeat domains of p100 and p105^15^. Recently, it has been demonstrated that CBS depletion enhances cisplatin efficacy by inhibiting NFκB activation^7^.

The results of the present study led us to identify CBS and NFκB as new molecular targets involved in cell response to 5-FU mediated by rpL3. We demonstrated that upon ribosomal stress induced by 5-FU, rpL3: (i) downregulates CBS protein levels; (ii) interacts with CBS and inhibits CBS half life; (iii) decreases NFκB activity by repressing its translocation to the nucleus; (iv) upregulates IκB-α protein levels by preventing its degradation. We finally demonstrated that rpL3 potentiates 5-FU efficacy inducing mitochondrial apoptotic pathway and inhibiting cell migration and invasion. These results indicate that rpL3 could be a promising adjuvant treatment in improving the efficacy of 5-FU based chemotherapy of lung cancer cells lacking functional p53.

## Results

### CBS and rpL3 expression profile in human normal and tumor tissues

**Figure 1:**
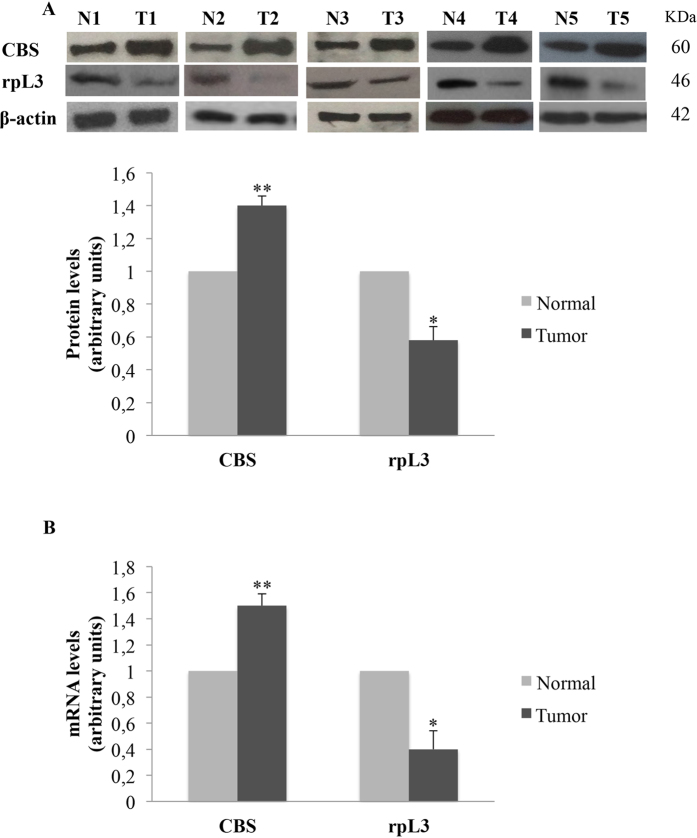
Expression of rpL3 and CBS in the lung tumor tissues. **(A)** Representative western blotting of CBS and rpL3 protein expression in human lung cancer specimens paired with the corresponding normal tissues. N:non-tumor tissue. T:primary tumor tissue. Each number corresponds to a case number. β-actin was used as loading control. Quantification of signals is shown. **P < 0.01, *P < 0.05 vs. normal tissues set at 1. **(B)** qRT-PCR data showing the expression of CBS and rpL3 mRNAs in tumor tissues. **P < 0.01, *P < 0.05 vs. normal tissues set at 1. Results illustrated in Figs 1–7 are representative of three independently performed experiments; error bars represent the standard deviation.

Emerging data suggest that CBS plays an important role in the regulation of cancer cell biology^5^. To evaluate CBS and rpL3 clinical significance in lung cancer, we employed quantitative real-time PCR (qRT-PCR) and immunoblotting to assess the expression of CBS and rpL3 at the mRNA and protein levels in 21 lung cancers and normal tissues. Comparison of human lung cancer specimens with patient-matched normal tissues revealed the up-regulation of CBS and the down-regulation of rpL3 in the cancers (Fig. 1A,B). Table 1 summarizes demographic, pathological and clinical data.

### rpL3 downregulates CBS expression upon 5-FU treatment

**Figure 2:**
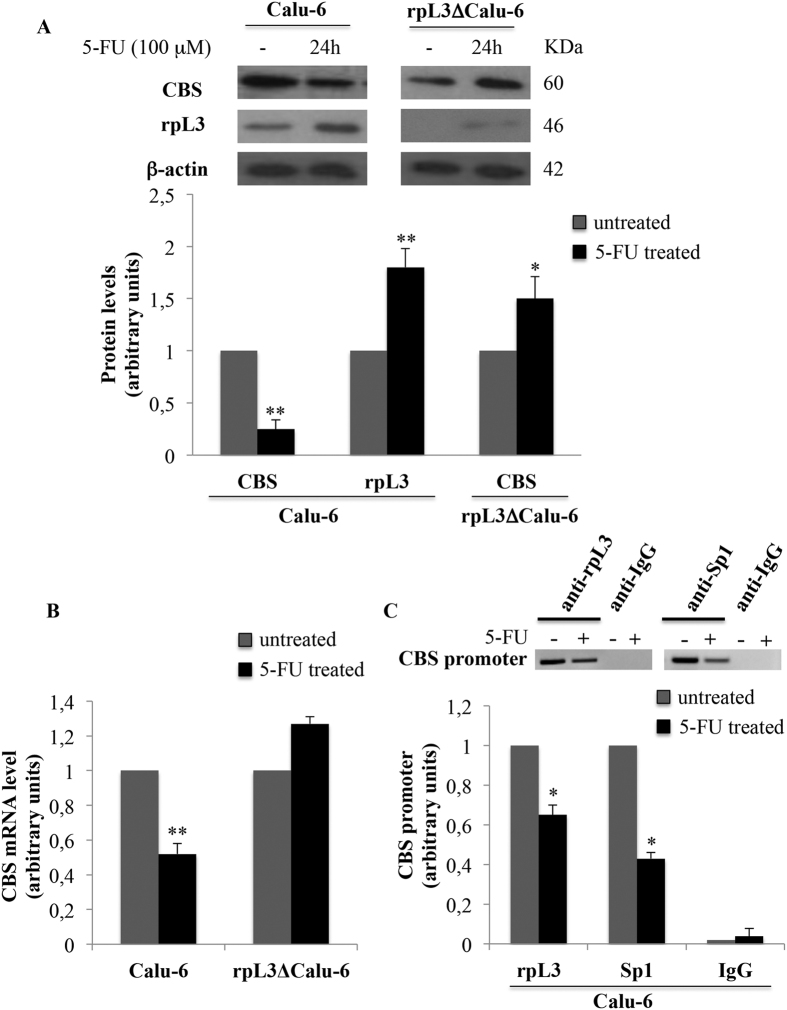
5-FU treatment is associated to the rpL3-mediated down-regulation of CBS at transcriptional level. **(A)** Representative western blotting of CBS protein expression. Calu-6 and rpL3ΔCalu-6 cells were treated with100 μM 5-FU for 24 h. Then, protein extracts were analyzed by western blotting assay with antibodies against CBS and rpL3. β-actin was used as loading control. Note that shRNA-mediated silencing of rpL3 abolished down-regulation of CBS after 5-FU treatment. Quantification of signals is shown. **P < 0.01, *P < 0.05 vs. untreated cells set at 1. **(B)** Total RNA from Calu-6 and rpL3ΔCalu-6 cells, untreated or treated with 100 μM 5-FU for 24 h, was subjected to qPCR with primers specific for CBS. Quantification of signals is shown. **P < 0.01, *P < 0.05 vs. untreated cells set at 1. **(C)** Analysis of the interaction between rpL3 and CBS gene promoter in response to 5-FU treatment. DNA-rpL3, DNA-Sp1 or DNA-IgG immunocomplexes from Calu-6 cells untreated or treated with 100 μM 5-FU for 24 h were subjected to qPCR with primers specific for CBS gene promoter. Quantification of signals is shown. *P < 0.05 vs. untreated Calu-6 cells set at 1

We have recently demonstrated that rpL3 is essential to mediate cell response to 5-FU in Calu-6 cells^4^. Having established the expression profile of CBS and rpL3 in tumors we became interested to investigate the possible involvement of rpL3 in the control of CBS expression in lung cancer treatment. Thus, we firstly assessed the intracellular levels of CBS in Calu-6 cells upon 5-FU treatment. To this aim, Calu-6 cells were treated with 100 μM of 5-FU for 24 h. After the treatment, the cells were lysated and proteins extracted analyzed by western blotting. The Fig. 2A shows that 5-FU treatment caused a decrease of CBS expression levels. Next, to investigate the role of rpL3, we performed analogous experiments in a cell clone namely rpL3ΔCalu-6 cells in which rpL3 is stably silenced^16^. In these cells, 5-FU failed to downregulate CBS expression levels (Fig. 2A). The same behavior occurs in a second independent clone namely rpL3ΔaCalu-6 cells (see Supplementary Fig. S1 on line, panel A).Changes of CBS protein amounts upon alteration in rpL3 expression levels and 5-FU treatment suggest that the down-regulation of CBS occurs through a molecular mechanism mediated by rpL3.

Since we have recently demonstrated that after drug treatment the ribosome-free rpL3 behaves as a transcription factor^4^,^16^, we became interested to determine if the rpL3-mediated down-regulation of CBS protein levels occurred via inhibition of CBS gene transcription. To this aim, total RNA from untreated and 5-FU treated Calu-6 and rpL3ΔCalu-6 cells was isolated and analyzed by quantitative RT-PCR using primers specific for CBS. We found a strong reduction of CBS mRNA levels in Calu-6 cells treated with 5-FU. Of note, no difference in CBS mRNA level was found in 5-FU treated rpL3ΔCalu-6 cells (Fig. 2B). Analogous experiments were performed in rpL3ΔaCalu-6 cells (see Supplementary Fig. S1 on line, panel B).These data indicate that rpL3 was necessary for regulating CBS mRNA levels in the cell response to 5-FU. Next, to test the presence of rpL3 on CBS gene promoter in the response to drug exposure, Calu-6 cells, untreated or treated with 100 μM of 5-FU for 24 h, were collected and subjected to Chromatin immunoprecipitation experiments by using anti-rpL3 antibodies, anti-IgG as negative control and anti-Sp1, a protein known to bind to the CBS promoter, as positive control.

The presence of rpL3 and Sp1 in DNA-immunoprecipitated complexes was assayed by western blotting (data not shown). qPCR assays on the samples were performed as previously reported^17^. Figure 2C shows that in untreated cells, rpL3 is able to bind CBS promoter. After 5-FU treatment, the binding of rpL3 on CBS promoter was significantly decreased compared to that observed in the control.

### rpL3 binds CBS and inhibits its stability

**Figure 3:**
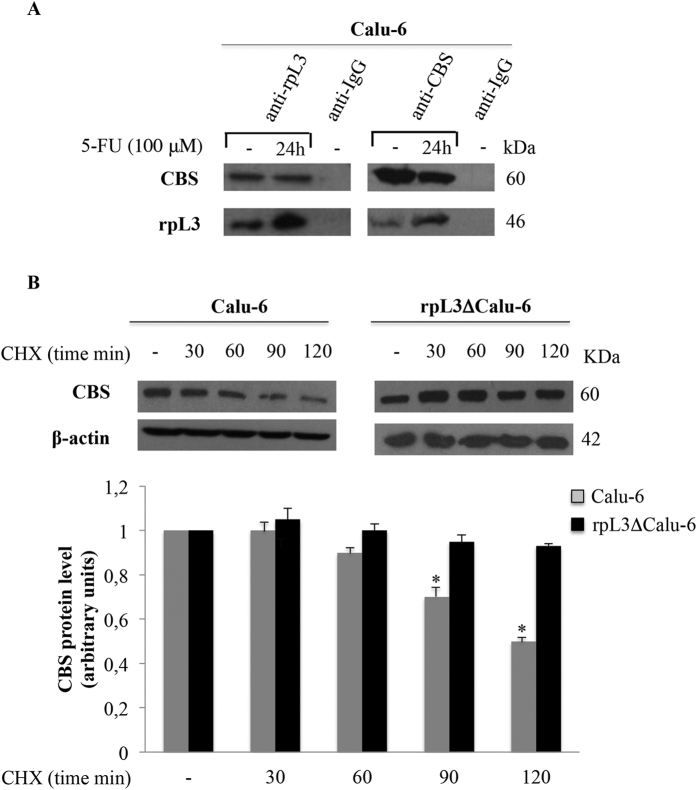
rpL3 binds CBS and negatively affects its half-life. (**A)** CBS and rpL3 were specifically immunoprecipitated from Calu-6 cell extracts with antibodies against the endogenous CBS and endogenous rpL3. Immunoprecipitates were separated by SDS–PAGE and immunoblotted with antibodies versus rpL3 and CBS. Note the absence of signal in IgG immunocomplex. **(B)** Calu-6 and rpL3∆Calu-6 cells were treated with CHX for 30, 60, 90 and 120 min. Then, cell lysates were immunoblotted with anti-CBS and anti-β-actin antibodies. Quantification of signals is shown. *P < 0.05 vs. untreated Calu-6 cells set at 1

We next investigated the possibility that rpL3 and CBS could associate *in vivo* by performing coimmunoprecipitation assays. To this aim, Calu-6 cells were treated with 100 μM of 5-FU for 24 h. Then, CBS and rpL3 were specifically immunoprecipitated from cell extracts by using antibodies against the endogenous proteins. Immunoprecipitated proteins were separated by SDS-PAGE and the presence of rpL3 and CBS was investigated by western blotting in the reciprocally immunoprecipitated complexes. The results of these experiments showed that rpL3 and CBS were co-immunoprecipitated (Fig. 3A). A control immunoprecipitate obtained with anti-IgG antibodies did not give any signal when probed with anti-CBS or anti-rpL3.

To understand the significance of rpL3 and CBS interaction, the turnover of CBS was determined in Calu-6 and rpL3∆Calu-6 cells by cycloheximide chase. Specifically, cells were incubated with cycloheximide for various times (30, 60, 90, 120 min). After the incubation, cells were harvested, lysated and the level of CBS was determined by western blot analysis (Fig. 3B). The results demonstrated that the loss of rpL3 was associated to an increase of CBS half-life. All together these data indicate that rpL3 physically interacts with CBS and induces its degradation.

### rpL3 regulates NFκB activation pathway upon 5-FU treatment

**Figure 4:**
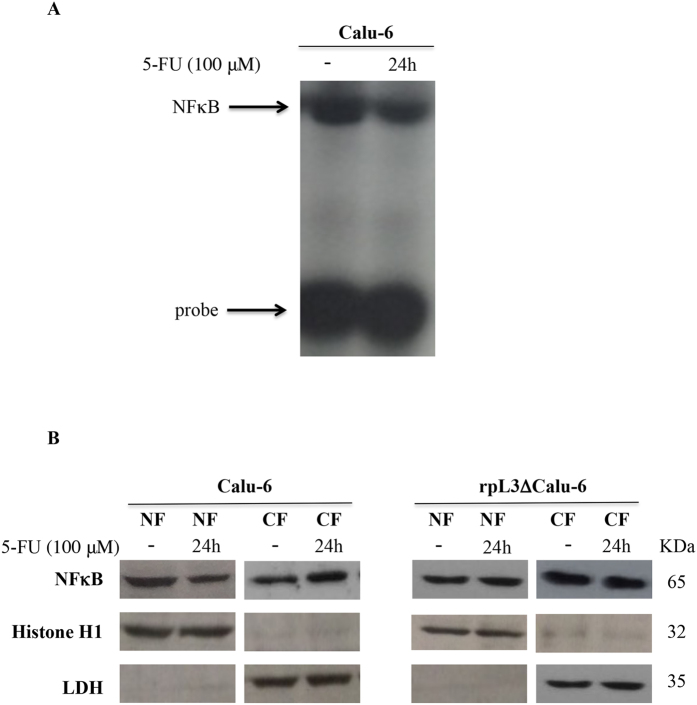
5-FU treatment inhibits NFκB DNA binding and decreases its nuclear translocation. **(A)** Calu-6 cells were incubated or not with 100 μM 5-FU for 24 h. Then, nuclear lysates were incubated with a ^32^P-labelled NFκB consensus probe and analyzed for NFκB DNA-binding complexes in EMSA. **(B**) Representative western blotting of NFκB expression. Calu-6 and rpL3∆Calu-6 cells were treated or not with 100 μM 5-FU for 24 h. After the treatment, cells were subjected to fractionation to obtain the nuclear fraction (NF) and the cytosolic fraction (CF). Protein extracts from the samples were analyzed by western blotting with antibodies against NFκB. Histone H1 and LDH were used as controls for NF and CF, respectively.

The NFκB pathway regulates apoptotic signals and has been implicated in the growth of several tumours including lung carcinoma^15^. Thus, to determine whether 5-FU treatment could interfere with NFκB activity, we performed EMSA using nuclear extracts from Calu-6 cells cultured in the presence of 100 μM 5-FU for 24 h. Briefly, the nuclear extracts were incubated with dsDNA oligonucleotides containing the NFκB recognition site. As shown in Fig. 4A, EMSA revealed constitutive binding of NFκB transcription factor in untreated Calu-6 cells. After 24 h, 5-FU treatment inhibited NFκB activation in Calu-6 cells. These data suggest that 5-FU is likely to interfere with the signalling cascade that leads to NFκB activation.

Next, to understand the molecular basis underlying NFκB activity in these cells, we examined the role of rpL3 in the control of nuclear translocation of NFκB protein after 5-FU treatment. To this aim, Calu-6 and rpL3ΔCalu-6 cells were treated with 100 μM 5-FU for 24 h. Then, protein extracts from the samples were analysed by western blotting. Consistent with EMSA results, the treatment of Calu-6 cells with 5-FU resulted in a significant decrease of the nuclear NFκB p65 protein (Fig. 4B). Of interest, in rpL3∆Calu-6 cells 5-FU treatment failed to inhibit NFκB translocation from cytoplasm to the nucleus.

### rpL3 stabilizes IκB-α in Calu-6 cells upon 5-FU treatment

**Figure 5:**
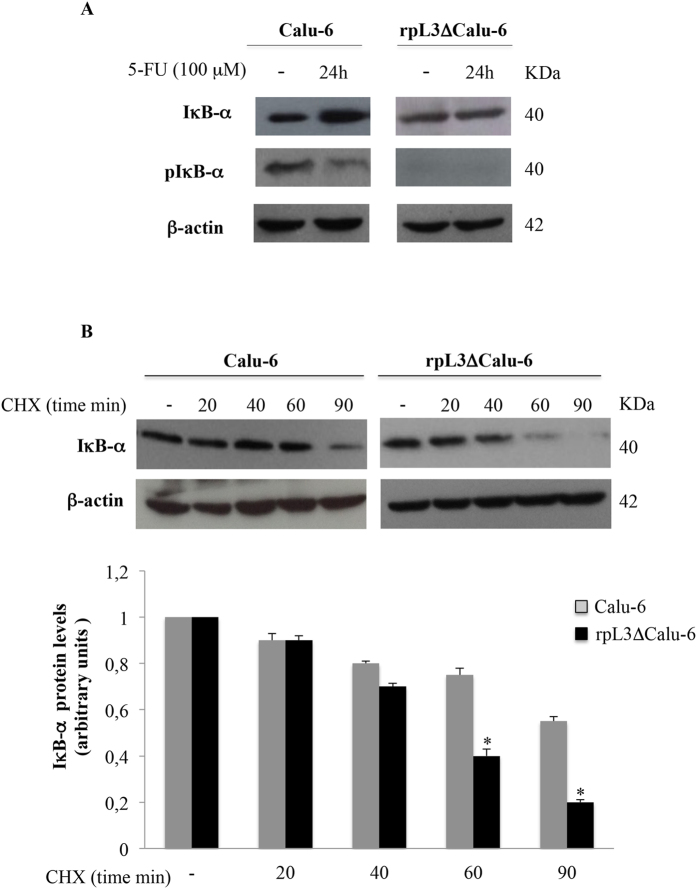
rpL3 prevents the degradation of IκB-α protein upon 5-FU treatment. (**A**) Representative western blotting of IκB-α and pIκB-α expression. Calu-6 and rpL3ΔCalu-6 cells were treated or not with 100 μM 5-FU for 24 h. After the treatment, protein extracts from the samples were analyzed by western blotting with antibodies against IκB-α, pIκB-α and β-actin as control. (**B**) Calu-6 cells and ∆rpL3Calu-6 cells were treated with CHX for 20, 40, 60 and 90 min. Then, cell lysates were immunoblotted with anti- IκB-α and anti-β-actin antibodies. Quantification of signals is shown. *P < 0.05 vs. untreated ∆rpL3Calu-6 cells set at 1.

The suppressed NFκB DNA binding activity could be due to a prevention of the degradation of IκB-α protein, which is an inhibitor of the canonical NF-κB pathway, upon 5-FU treatment^18^. To test this hypothesis, Calu-6 and rpL3ΔCalu-6 cells were treated with 100 μM 5-FU for 24 h. Immunoblot analysis of cell extracts revealed significant changes (about two fold induction) in protein levels of IκB-α associated to a strongly reduction of pIκB-α in 5-FU Calu-6 cells (Fig. 5A). Of note, this effect was not observed in 5-FU treated rpL3∆Calu-6 cells indicating that the effect of 5-FU on IκB-α expression was specifically mediated by rpL3. In conclusion, the above results suggest that 5-FU inhibits NFκB activity preventing IκB-α degradation in rpL3-dependent manner. To investigate the mechanism by which rpL3 increases IκB-α levels, we next determined the effect of rpL3 on IκB-α protein stability.

The degradation of IκB-α was evaluated by treating Calu-6 and rpL3∆Calu-6 cells with cycloheximide for different times (0, 20, 40, 60, 90 min). The results demonstrated that silencing of rpL3 was associated to a decrease of IκB-α half-life (Fig. 5B). These data indicate that rpL3 increases IκB-α protein stability.

### Role of rpL3 in mitochondrial apoptosis upon 5-FU treatment

**Figure 6:**
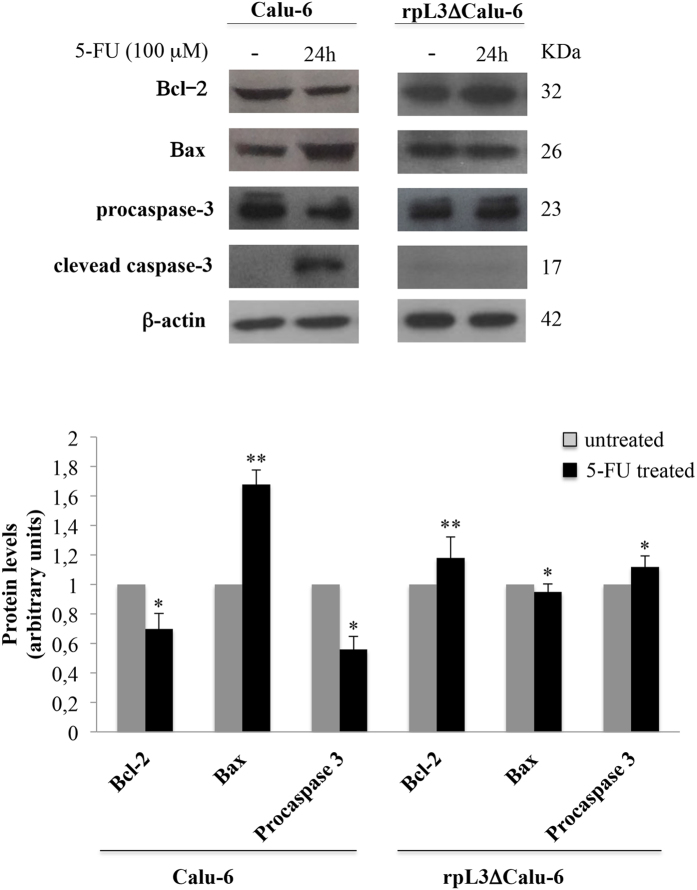
Role of rpL3 on apoptosis. Representative western blotting of Bcl-2, Bax, procaspase-3 and clevead caspase-3 protein expression. Calu-6 and rpL3ΔCalu-6 cells were treated or not with 100 μM 5-FU for 24 h. After the treatment, protein extracts from the samples were analyzed by western blotting with indicated antibodies. Quantification of signals is shown. **P < 0.01, *P < 0.05 vs. untreated Calu-6 cells set at 1.

As previously documented, NFkB protein can act as survival factor by inhibiting apoptosis^19^ and the downregulation of CBS triggers mitochondrial apoptosis^20^. Recently, we have shown a proapoptotic function of rpL3^17^. Starting from these findings, in order to investigate the factors leading to the rpL3-induced apoptosis in Calu-6 cells upon 5-FU treatment, we determined the effect of 5-FU-induced rpL3 on activities of apoptosis-related proteins of the mitochondria-mediated pathway. In particular, the ratio of Bcl-2/Bax was used to evaluate the occurrence and severity of apoptosis^21^. To this aim, Calu-6 cells and rpL3ΔCalu-6 cells were treated with 100 μM 5-FU for 24 h. Then, cells were lysated and protein extracts were analysed by western blotting for the expression profile of Bax and Bcl-2. The Fig. 6 shows a marked decrease of Bcl-2 levels associated to an increase of Bax levels in Calu-6 cells treated with 5-FU compared with untreated cells. Of interest, these effects were not obeserved when we treated rpL3∆Calu-6 cells with 5-FU. Among caspase family, which are activated when the ratio of Bcl-2/Bax is reduced, we analyzed the expression of caspases-3 which is a crucial regulator in the caspase-dependent cell apoptosis pathway^22^. As attended, the active caspase-3 form, which is proteolytically generated during apoptosis, was observed only in 5-FU treated Calu-6 cells. This data revealed that the involvement of a caspase-dependent pathway may lead to rpL3-mediated apoptosis.

### rpL3 potentiates the citotoxic activity of 5-FU in Calu-6 cells

**Figure 7:**
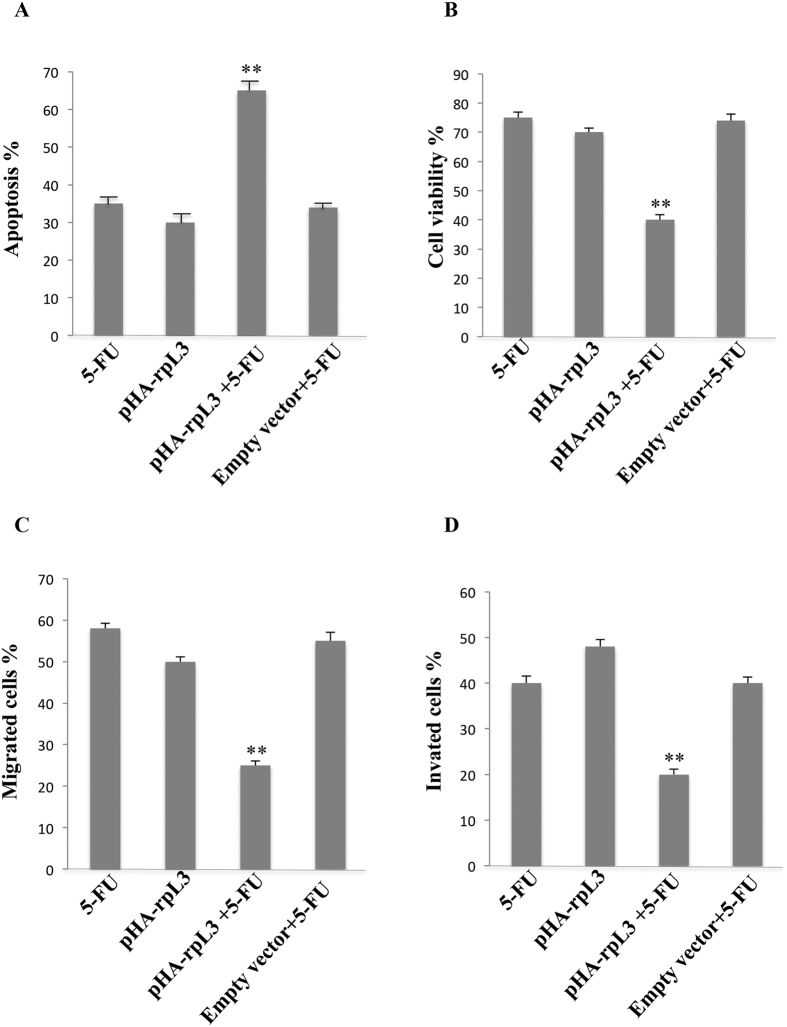
Role of rpL3 on cell viability, migration and invasion upon 5-FU treatment. Calu-6 cells were transiently transfected with pHA-rpL3 and treated with 100 μM 5-FU for 24 h or untreated. Then, cell viability was evaluated using the **(A)** TMRE and **(B)** MTT assays**. (C)** Overexpression of rpL3 inhibits cell migration upon 5-FU treatment. Calu-6 cells were transiently transfected with pHA-rpL3 and treated with 100 μM 5-FU for 24 h or untreated. Then migration of cells were examined using Boyden chamber. **(D**) Overexpression of rpL3 inhibits cell invasion of fibronexin matrix by Calu-6 cells upon 5-FU treatment. Invasion of cells through fibronexin matrix was examined using Boyden chamber. Quantification of signals is shown, **P < 0.01 vs. 5-FU treated Calu-6 cells.

Our results prompted us to investigate whether the up-regulation of rpL3 may potentiate the cytotoxic effect of 5-FU. With the aim of examining the combined effect of rpL3 and 5-FU on the cell proliferation, Calu-6 cell viability was evaluated by using TMRE and MTT assays. Specifically, Calu-6 cells were transiently transfected with 2 μg of pHA-rpL3 or empty vector. Then, cells were treated with 100 μM 5-FU. 24 h later, modifications of mitochondrial inner membrane were estimated by TMRE staining and analysed by flow cytometry. As expected, rpL3 or 100 μM 5-FU treatment induced apoptosis on these cells (Fig. 7A). Of note, in cells transfected with pHA-rpL3 and treated with 5-FU the cytotoxicity was increased of about 30% as compared with cells treated with 5-FU or rpL3 alone (Fig. 7A). As shown in Fig. 7B the results obtained with MTT assay were in line with those obtained with the TMRE assay.

Furthermore, we investigated the role of rpL3 overexpression alone or in combination with 5-FU on Calu-6 cell migration and invasion. To this purpose, Calu-6 cells, transiently transfected with pHA-rpL3, treated or not with 100 μM of 5-FU for 24 h, were analyzed by using Boyden chamber migration assay. As shown in Fig. 7C and Supplementary Fig. S2 on line, the migration ability of 5-FU treated Calu-6 cells was reduced of about 40% as compared with untreated cells set as 100%. When rpL3 was overexpressed, the migration ability of 5-FU treated Calu-6 cells was further reduced (75%) demonstrating that rpL3 overexpression was able to improve 5-FU-mediated inhibition of cell motility. We also examined whether rpL3 affected the invasion ability of Calu-6 cells. A significant decrease in invasion ability of Calu-6 cells was observed when these cells were transiently transfected with pHA-rpL3 and treated with 5-FU as compared to untrasfected, 5-FU treated cells (see Supplementary Fig. S2). Quantification of invading cell numbers indicated that overexpression of rpL3 reduced the invasion ability by 80% (Fig. 7D).

All together these results strongly suggest that the ectopic expression of rpL3 allowed a more potent cytotoxic activity of 5-FU on lung cancer cells.

## Discussion

Several evidences indicate that perturbation in the expression of rp induces the tumorigenesis^23^. Rp have been found overexpressed in cancer, for example rpL19, rpL7a, rpL37 in prostate cancer^24^ rpL15 and rpL19 in gastric cancer^25^,^26^. The expression of other rp was found reduced as rpL27, rpL37, rpL41 in a subset of cell lines derived from nasopharyngeal carcinomas^27^. Changes in the expression levels of rps have been used as prognostic or predictive values to distinguish between normal and cancer cells. To date, increased expression of the rpL19 accurately identifies prostatic malignancy, can discriminate the progressive form of the disease and predicts the biological behavior of individual prostate cancers and patient survival^25^. Decreased expression of rpL15 may serve as a prognostic marker in pancreatic ductal adenocarcinoma and was significantly associated with poor overall survival^28^.

Some rps participate the tumorigenesis by their extraribosomal functions by regulating the expression of oncogene and tumor suppressors.

A number of rps including rpL37, rpS15, rpS20 have been shown to bind MDM2 and inhibit its E3 ligase activity, leading to p53 stabilization and cell cycle arrest^29^.

Ribosome biogenesis is one of the major biosynthetic activities in a cancer cell and thus represents a good target in anticancer therapy. Different levels of perturbation of ribosome biogenesis by chemotherapeutic drugs have been defined, including inhibition of rRNA gene transcription, inhibition of early and late maturation of rRNA precursors, and perturbation linked to disintegration of nucleolar structures^30^.

Emerging data are accumulating about the identification of other targets in the ribosome biogenesis machinery including ribosomal proteins themselves. rpS2 was reported to be a therapeutic targeting for the eradication of prostate cancer in preclinical tumor modeling studies^31^. Silencing of rpL19 abrogated the aggressive phenotype of human prostate cancer^32^.

Lung cancer represents one of the most common malignant tumors in the world and the leading cause of cancer-related death in many countries^33^. 5-FU-based combination therapies have been for a long time standard treatments; however resistance to 5-FU has been recognised as the main reason for cancer therapy failure. Therefore, attempts to enhance the therapeutic effectiveness of this drug are needed in order to improve patients survival. To find more appropriate therapeutic opportunities for cancer treatment, the identification of new molecular targets involved in cell response to 5-FU treatment may be the basis for new combination therapies. We have recently demonstrated that human. rpL3 acts as stress sensing molecule essential in the cell response to ribosomal stress caused by 5-FU in cancer cells lacking active p53^4^,^34^. Specifically, we have demonstrated that after 5-FU treatment rpL3 is up-regulated and accumulates as ribosome free form needed to mediate 5-FU apoptotic cell response. In fact the loss of rpL3 makes chemotherapeutic drug ineffective. We have demonstrated that ectopic expression of rpL3 in cancer cells induces cell cycle arrest or apoptosis by positively modulating p21 expression at transcriptional and post-translational levels^16^,^17^. Moreover, we found that rpL3 status was associated to chemoresistance since the loss of rpL3 makes chemotherapeutic drugs, such as 5-FU, oxaliplatin, actinomycin D and cisplatin ineffective^4^,^16^,^35^.

It has been recently reported a functional relation between H_2_S and p21 expression. In particular, an increase of H_2_S amount into the cells is associated to down-regulation of p21 expression leading to cancer proliferation^36^. Furthermore, several lines of evidence from previous studies reported increased expression of CBS in various cancers^5^. In the light of these findings we hypothesized the existence of a new rpL3 mediated pathway in response to 5-FU treatment involving CBS. We employed qRT-PCR and immunoblotting to assess the expression of CBS at mRNA and protein levels respectively. We found that the expression of CBS is higher in lung tumors than in normal tissues confirming the oncogenic potency of CBS. Analysis of expression profile of rpL3 in the same tissues reveals that rpL3 is expressed at lower level in tumors than in normal tissues (Fig. 1). Furthermore, we demonstrated that 5-FU treatment is associated to changes of CBS expression levels in Calu-6 cells. At 24 h of treatment, we observed a marked downregulation of CBS at protein and mRNA levels (Fig. 2A,B). Of note, silencing of rpL3 abolishes these effects indicating that the alteration of CBS levels after drug treatment is rpL3-dependent. Results of ChIP experiments imply a negative role of rpL3 in CBS gene transcription (Fig. 2C). Analysis of immunoprecipitates of rpL3 in Calu-6 cells shows that rpL3 and CBS coimmunoprecipitate together indicating that these proteins associate *in vivo* (Fig. 3A). These results led us to hypothesize that the specific interaction between rpL3 and CBS could affect CBS turnover. We demonstrated that in cell stably depleted of rpL3, the half life of CBS was decreased (Fig. 3B). These data demonstrate that rpL3 acts as a negative regulator of CBS stability. On this basis, we propose that upon ribosomal stress induced by 5-FU, ribosome free rpL3 represses CBS expression acting as repressor of transcription and as negative regulator of protein stability. Taken together these results introduce rpL3 as an important player in CBS activity.

It is well established a strong correlation between cancer and inflammation^37^. A plausible hypothesis is that CBS up-regulation observed in human tumor tissues and cancer cells could be a consequence of local or systemic inflammation^38^. NFκB is a key factor with a role as a pivotal link between inflammation and cancer^39^. There is considerable evidence that NFκB is constitutively activated in a variety of solid tumors, including lung cancer^40^. It has been shown that 5-FU suppresses NFκB expression in human salivary gland cancer cells^41^. Furthermore, numerous studies in cancer cells have demonstrated that inhibition of NFκB signaling with various approaches increase the efficacy of chemotherapeutics *in vitro* and *in vivo*^42^,^43^.

These findings prompted us to investigate whether in condition of 5-FU treatment rpL3 status could induce pertubation in NFκB signaling. Our data indicated a strong correlation between intracellular levels of rpL3 and the activation of NFκB signaling (Fig. 4). In particular, our results show that rpL3 silencing induces a strong increase in IκB-α half life (Fig. 5) with consequent down-regulation of NFκB signaling. These data together with recent finding demonstrating that silencing of CBS in ovarian cancer cells is associate to a decrease of NFκB promoter activity^7^, led us to suppose that the drastic reduction in NFκB activity, observed after 5-FU treatment in Calu-6 cells, is a result of rpL3 mediated CBS down-regulation and IκB-α stabilization. In summary, our study identify rpL3 as a protein that targets either CBS to destabilize it acting at transcriptional and post-translational levels or IκBα acting at post-tanslational level with consequent reduction of NFκB activity. Numerous studies in cancer cells have demonstrated that the inhibition of either NFκB or CBS increase the efficacy of chemioterapeutic drugs^7^,^43^. As a newly identified CBS and NFκB repressor, we suggest that rpL3 could be used in association with 5-FU to enhance the susceptibility of lung cancer cells to this drug. In fact, we report that co-treatment of cells with 5-FU and pHA-rpL3 lead to a markedly increased sensitivity of cells to 5-FU. In particular, our results demonstrate that rpL3 strongly increase 5-FU mediated cytotoxic effect (Figs 6 and 7). In addition, when cells were treated with a combination of 5-FU and pHA-rpL3 we obserbed a strong reduction in cell migration and invasion (Fig. 7). These data indicate that rpL3 overexpression associates to a strong increase of drug cytotoxicity. Taking together these results show that the efficacy of 5-FU chemotherapy depends on rpL3 status. Along this line, the knowledge of rpL3 status in p53 null cancers may have a significant value in terms of the efficacy of chemotherapy based on 5-FU. Hence, the development of treatments aimed at upregulating rpL3 may be beneficial for the treatment of cancers lacking p53 and downregulated rpL3.

## Methods

### Tissues

The research has been carried out in accordance with the Declaration of Helsinki (2013) of the World Medical Association. Informed consent was obtained from all 21 subjects involved in this study and all methods described in this section were carried out in accordance with the guidelines approved by the ethics committee of the National Cancer Institute ‘G. Pascale’ of Naples. Homogenization of tissues, was performed as previously reported^44^. Total RNA was extracted once from the tissue samples by using an ultrapure TRIzol reagent (GibcoBRL, Carlsbad, CA, USA) and used in three independent experiments.

### Cell cultures, DNA, transfections and drug treatments

Calu-6 cells, rpL3ΔCalu-6 and rpL3ΔαCalu-6 derived from Calu-6 cell line and stably silenced for rpL3^16^, were cultured in Dulbecco’s Modified Eagle’s Medium (DMEM) with glutamax (Invitrogen, Carlsbad, California) supplemented with 10% fetal bovine serum (FBS), 2 mM L-glutamine, penicillin-streptomycin 50 U/ml and 0.5 μg/ml puromycin (Sigma-Aldrich).

Drug treatments were performed by adding to cells 100μM 5-FU for 24 h. Transfection of pHA-rpL3 plasmid was performed in cells using Lipofectamin 2000 as previously described^45^.

### Realtime PCR

Total RNA was isolated from cells using RNeasy Plus Mini kit (QIAGEN). RNA was first retrotranscribed as previously reported^44^ and then realtime PCR was carried out using TaqManH SYBR Green Master Mix (Applied Biosystems). The primers for human CBS (PPH13484B-200) and β actin (PPH00073G-200 ACTB) were from QIAGEN. The comparative C_t_ method was used to calculate the relative abundance of the mRNA and compared with that of β-actin expression^46^,^47^.

### Chromatin immunoprecipitation

Chromatin immunoprecipitation assay was performed as previously reported^16^. The sequence for primers used to amplify CBS promoter were: Forward 5′-CGCCCCTCTTTTCCATGTATCCGTCCAG-3′ and Reverse 5′-ACCTGGCATT- GGTGGGCGTCCTCACA-3′.

### Immunoprecipitation and western blotting

Immunoprecipitation assay was performed as previuosly reported^45^. Calu-6 whole cell lysate (1 mg) was incubated with 30 μl of protein A/G agarose beads coated with 5 μg of anti-CBS (Santa Cruz Biotechnology) or anti-rpL3 (Primm, Milan, Italy), at 4 °C for 12 h. The eluted proteins were loaded on 12% SDS-PAGE and detected by western blotting. Aliquots of protein samples (30 μg) were resolved by 12% SDS-gel electrophoresis and transferred into nitrocellulose filters. The proteins were visualized with enhanced chemiluminescence detection reagent according to the manufacturer’s instructions (Pierce, Rockford, Illinois).

Western blotting analysis was performed as previously reported^48^. The membranes were challenged with anti-rpL3 (Primm, Milan, Italy), anti-CBS, anti-NFκB, anti-IκB-α, anti-pIκB-α and anti-β-actin (Santa Cruz Biotechnology). Proteins were visualized with enhanced chemiluminescence detection reagent according to the manufacturer’s instructions (Pierce, Rockford, Illinois).

### Protein half-life analynsis

Cells were treated with Cycloheximide (CHX,Sigma-Aldrich, St. Louis, MO, USA) 0,1 mg/ml for different times, and subsequently harvested and lysed using RIPA lysis buffer (50 mM Tris-HCl pH 7.4, 1% NP40, 0,5% Na-deoxycolate, 150 mM NaCl, 1 mM Na3VO4, 1 mM NaF, 1X EDTA-free Roche protease inhibitor cocktail). Protein extracts from samples were analyzed by western blotting.

### Preparation of nuclear extracts and electrophoresis mobility shift assay

Nuclear extracts was performed as previously reported^49^,^50^, EMSA assay was performed as previously reported^51^.

### Mitochondrial membrane potential measurement

To quantify changes in mitochondrial membrane potential cells were labeled with 50 nM of the mitochondrial membrane potential-sensitive fluorescent dye TMRE (Invitrogene) for 30 mi a 37 °C, analyzed by a Cyan-ADP Flow Cytometer (DAKOCytomation) and quantified using Summit software.

### MTT assay

MTT assay was performed as previously described^52^.

### Boyden chamber migration and invasion

Starved Calu-6 and rpL3∆Calu-6 cells (1 × 10^5^ cells) were plated into transwell chamber in serum free D-MEM medium. The lower chambers of the plate were supplied with D-MEM medium supplemented with 10% FBS. Then migration of cells was examined using Boyden chamber. 24 h after indicated treatments cells were fixed and stained with crystal violet and counted under microscope. For cell invasion assay Boyden chamber equipped with membranes precoated with fibronectin were used^53^.

### Statistical analysis

Error bars represent mean ± s.d. from n = 3 biological replicates. *P < 0.05 was considered significant, **P < 0.01 was considered highly significant; Student t-test is used throughout.

## Additional Information

**How to cite this article**: Russo, A. *et al*. rpL3 promotes the apoptosis of p53 mutated lung cancer cells by down-regulating CBS and NFκB upon 5-FU treatment. *Sci. Rep.* **6**, 38369; doi: 10.1038/srep38369 (2016).

**Publisher's note:** Springer Nature remains neutral with regard to jurisdictional claims in published maps and institutional affiliations.

## Supplementary Material

Supplementary Information

## Figures and Tables

**Table 1:** ADC: adenocarcinoma; na: not available.

Case no.	Age (years)/Sex (M/F)	Site		Diagnosis
		Primary	Relapse Tissue	
1	77/M	lung	none	ADCmix (signet, solid, acinar) grade III
2	59/M	lung	brain	ADCmix (mucinous, solid) grade II
3	62/F	lung	none	ADCmix (lepidic, acinar) grade I
4	66/M	lung	none	ADCmix (signet, solid, lepidic) grade II
5	58/M	lung	none	Adenosquamous grade III
6	57/M	lung	none	ADCmix (solid, papillary, acinar) grade III
7	75/M	lung	none	ADCmix (acinar, solid, lepidic) grade III
8	64/M	lung	none	ADC mucinous grade II
9	64/M	lung	linfoma	ADC solid grade III
10	57/M	lung	lung	ADC papillary grade II
11	62/M	lung	na	ADCmix (lepidic, acinar, solid) grade II
12	48/F	lung	na	ADCmix (solid, acinar, papillary) grade II
13	57/M	lung	na	ADCmix (papillary, lepidic, acinar) grade II
14	66/F	lung	na	ADCmix (solid, signering, papillary) grade III
15	72/F	lung	na	ADCmix (papillary, micropap, acinar) grade II
16	64/M	lung	na	ADCmix (acinar, papillary, solid) grade II
17	33/F	lung	na	Adenoid cystic carcinoma
18	69/F	lung	none	Adenosquamous grade II
19	72/M	lung	bone	ADC lepidic grade I
20	68/M	lung	brain	Squamous grade II
21	48/M	lung	none	ADC lepidic grade I
