# Supplementary material for: rpL3 promotes the apoptosis of p53 mutated lung cancer cells by down-regulating CBS and NFκB upon 5-FU treatment
Source: Sci Rep. 2016 Dec 7;6:38369. doi: 10.1038/srep38369 (PMC5141482; doi:10.1038/srep38369)
Supplement: Supplementary Information [file srep38369-s1.pdf]

rpL3 promotes the apoptosis of p53 mutated lung cancer cells by down-regulating CBS and NFkB upon 5-FU treatment

Annapina Russo[1]*, Assunta Saide[1], Roberta Cagliani[1], Monica Cantile[2], Gerardo Botti[2], Giulia Russo[1]

[1]Department of Pharmacy, University of Naples "Federico II", Via Domenico Montesano 49, 80131 Naples, Italy
[2]Department of Pathology, Istituto Nazionale Tumori "Fondazione Pascale"-IRCCS via Mariano Semmola, Napoli 80131, Italy

*Correspondence to: Annapina Russo, Department of Pharmacy, University of Naples "Federico II", Via Domenico Montesano 49, 80131 Naples, Italy, Tel: +39 081 678414; +39 081 7463074; Email: annapina.russo@unina.it

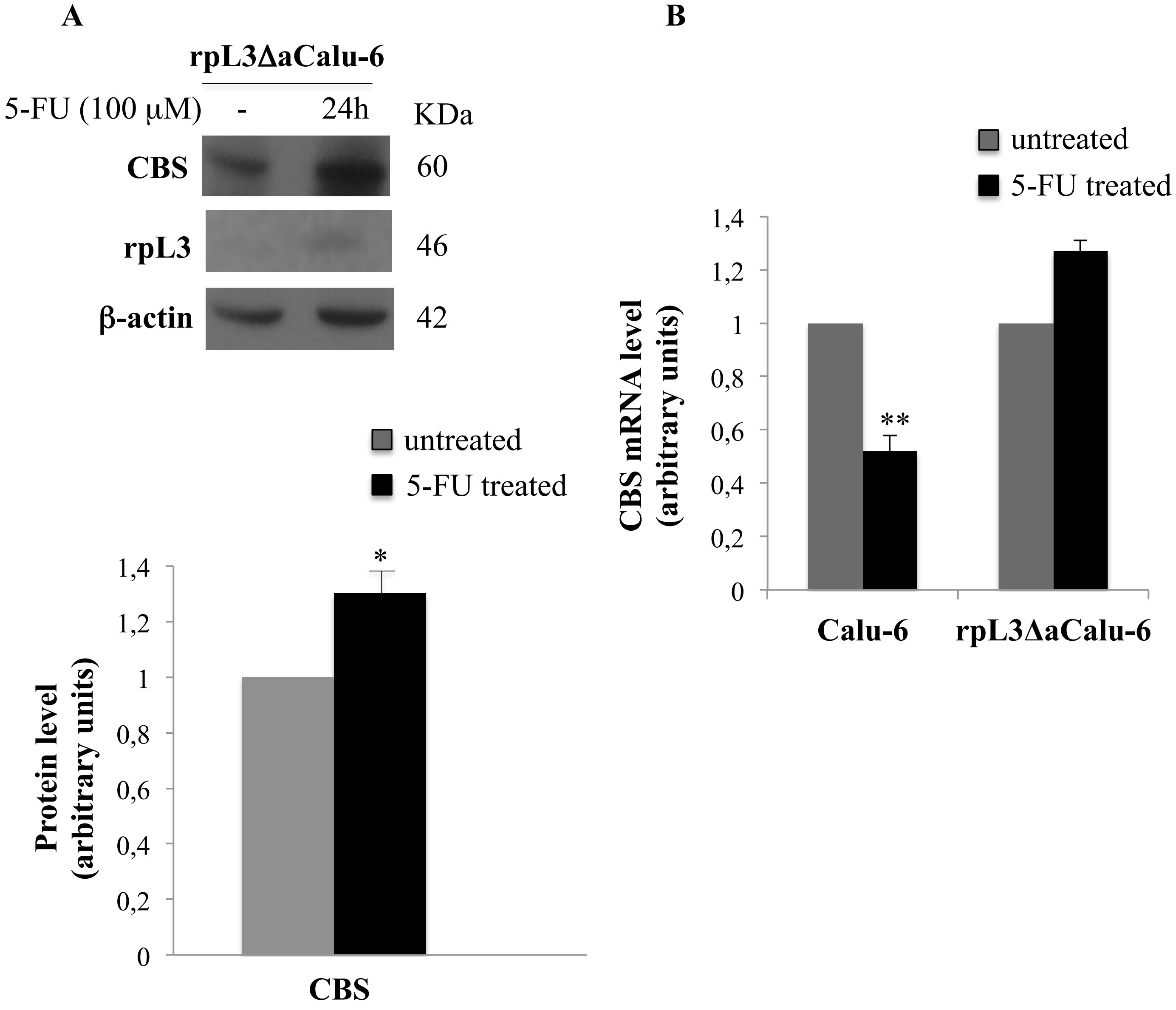

Supplementary Figure S1. 5-FU treatment is associated to the rpL3-mediated down-regulation of CBS at mRNA and protein levels. (A) Representative western blotting of CBS protein expression. rpL3ΔaCalu-6 cells (another cell clone) were treated with100 μM 5-FU for 24 h. Then, protein extracts were analyzed by western blotting assay with antibodies against CBS and rpL3. β-actin was used as loading control. Note that shRNA-mediated silencing of rpL3 abolished down-regulation of CBS after 5-FU treatment. **(B)** Total RNA from Calu-6 and rpL3ΔaCalu-6 cells, treated with 100 μM 5-FU for 24 h, was subjected to qPCR with primers specific for CBS. Quantification of signals is shown. **P<0.01, *P<0.05 vs.untreated Calu-6 cells set as 1.

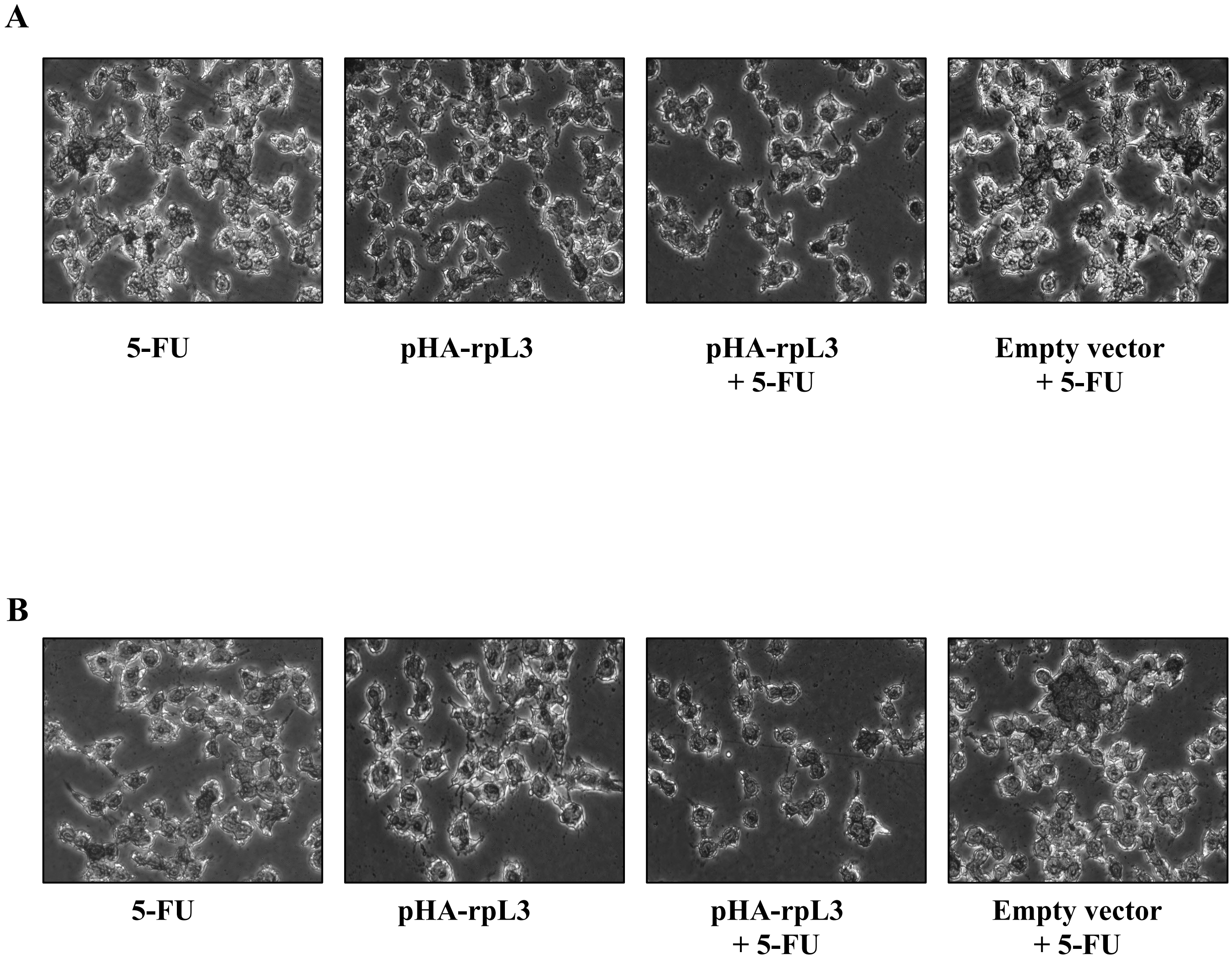

Supplementary Figure S2. Role of rpL3 on cell migration and invasion upon 5-FU treatment. Representative images of cell migration **(A)** and invasion **(B)** assays in Calu-6 cells. Calu-6 cells were transiently transfected with pHA-rpL3 or empty vector and treated with 100 μM 5-FU for 24 h or untreated. Then migration and invasion ability of cells were examined using Boyden chamber.
